# The p7 Protein of Hepatitis C Virus Forms Structurally Plastic, Minimalist Ion Channels

**DOI:** 10.1371/journal.pcbi.1002702

**Published:** 2012-09-20

**Authors:** Danielle E. Chandler, François Penin, Klaus Schulten, Christophe Chipot

**Affiliations:** 1 Department of Physics and Beckman Institute, University of Illinois at Urbana-Champaign, Urbana, Illinois, United States of America; 2 Bases Moléculaires et Structurales des Systèmes Infectieux, IBCP, Université Lyon 1, Univ Lyon, France; CNRS, UMR 5086, Lyon, France; 3 Department of Physics and Beckman Institute, University of Illinois at Urbana-Champaign, Urbana, Illinois, United States of America; 4 Beckman Institute, University of Illinois at Urbana-Champaign Urbana, Illinois, United States of America; 5 Équipe de Dynamique des Assemblages Membranaires UMR 7565, Université de Lorraine, Vanduvre-lès-Nancy, France; Wellcome Trust Sanger Institute, United Kingdom

## Abstract

Hepatitis C virus (HCV) p7 is a membrane-associated oligomeric protein harboring ion channel activity. It is essential for effective assembly and release of infectious HCV particles and an attractive target for antiviral intervention. Yet, the self-assembly and molecular mechanism of p7 ion channelling are currently only partially understood. Using molecular dynamics simulations (aggregate time 1.2 *µ*s), we show that p7 can form stable oligomers of four to seven subunits, with a bias towards six or seven subunits, and suggest that p7 self-assembles in a sequential manner, with tetrameric and pentameric complexes forming as intermediate states leading to the final hexameric or heptameric assembly. We describe a model of a hexameric p7 complex, which forms a transiently-open channel capable of conducting ions in simulation. We investigate the ability of the hexameric model to flexibly rearrange to adapt to the local lipid environment, and demonstrate how this model can be reconciled with low-resolution electron microscopy data. In the light of these results, a view of p7 oligomerization is proposed, wherein hexameric and heptameric complexes may coexist, forming minimalist, yet robust functional ion channels. In the absence of a high-resolution p7 structure, the models presented in this paper can prove valuable as a substitute structure in future studies of p7 function, or in the search for p7-inhibiting drugs.

## Author Summary

Hepatitis C remains a serious global health problem affecting more than 2% of the world's population, and current therapies are effective in only a subset of patients, necessitating an ongoing search for new treatments. The p7 viroporin is considered to be an attractive possible drug target, but rational drug design is hampered by the absence of a high-resolution p7 structure. In this paper, we explore possible structures of oligomeric p7 channels, and discuss the strengths and shortcomings of these models with respect to experimentally determined properties, such as pore-lining residues, ion conductance, and compatibility with low-resolution electron microscopy images. Our results present an image of p7 as a rudimentary, minimalistic ion channel, capable of existing in multiple oligomeric states but exhibiting a bias towards hexamers and heptamers. We believe that the work presented here will be valuable for future research by providing plausible 3-dimensional atomic-resolution models for the visualization of the p7 viroporin and serve as a basis for future computational studies.

## Introduction

Hepatitis C virus (HCV) infection is a global health problem affecting approximately 2% of the world's population [1], [2]. HCV is a leading cause of chronic hepatitis, liver cirrhosis and hepatocellular carcinoma, and current therapies based on pegylated interferon and ribavirin are poorly tolerated by patients and ineffective in up to 50% of cases [3]. Among the difficulties in treating HCV is its high degree of genetic diversity — i.e., there are seven distinct genotypes of HCV, each with many subtypes [4], which respond differently to treatment [5]–[7]. This genetic diversity is due in part to the error-prone RNA polymerase of HCV, along with its rapid replication rate; the same features can lead to further viral diversification within individual patients, increasing resistance to treatment [8]–[10]. HCV drug design must, therefore, target characteristics that are well-conserved among all HCV genotypes, as well as prove robust against mutations that could confer viral resistance. The assembly process and ion-channel function of the p7 viroporin, now known to be essential for viral replication, constitute potential drug targets. For example, the small molecule BIT225, which is thought to inhibit the ion-channel activity of p7, has shown promising results in recent clinical trials [11], though its mechanism of action is not yet well understood. Such cases highlight the biomedical relevance of developing functional models of the p7 channel.

HCV, first identified in 1989 [12], [13], is a member of the family *Flaviviridae*, which is a class of small enveloped RNA viruses. The HCV genome consists of a 9.6-kb positive-stranded RNA molecule encoding a single polyprotein precursor, later processed by both host and viral proteases into ten separate proteins. These are: the Core protein, which composes the nucleocapsid; the viral envelope E1 and E2 glycoproteins; the p7 viroporin; NS2, required for virus assembly; and the replication machinery consisting of NS3, NS4A, NS4B, NS5A, and NS5B (reviewed in [14]).

The focus of the present work is on p7, a small integral membrane protein of 63 amino acids, which oligomerizes [15]–[18], forming cation-selective pores [15], [16], [19]–[22]. It has been demonstrated that, while dispensable for RNA replication, p7 is essential for efficient HCV infectivity *in vivo* [23] and the production of infectious virus particles [24], [25]. Most recently, it has been shown that p7 ion channel activity, resulting in a global loss of organelle acidity in the host cell, is required for the effective assembly and release of nascent virions [26]. Furthermore, there is a growing body of evidence suggesting that p7 is critical for other functions in virus assembly and egress unrelated to its channel activity (reviewed in [27]), and that it likely acts in concert with additional viral factors such as Core, E1, E2 and NS2 [24], [28]–[33].

The ability of p7 to control the permeability of the membrane to ions and to facilitate virus production qualifies it as a viroporin, alongside with, for instance, M2 from influenza A virus [34], [35], picornavirus 2B [35], [36] and Vpu from HIV-1 [37], [38]. The essential features of a viroporin are that it forms small membrane-spanning structural units, usually consisting of one or two helices that can oligomerize into a channel [35], [38], [39]. The resulting structures range in complexity from passive, non-selective pores, which allow ions to flow through in an uncontrolled fashion, to selective ion channels with a specific gating mechanism, as found, for example, in M2. Weakly selective ion channels like Vpu fall somewhere in the middle, exhibiting “channel-pore dualism” [37], [40], [41].

The p7 protein of HCV forms two antiparallel transmembrane (TM) segments connected by a conserved, cytosolic, positively charged loop region, with both the N– and C–termini facing the lumen of the endoplasmic reticulum (ER) [42]. Combined NMR experiments and molecular dynamics (MD) simulations that we published recently [16], [43] led to the identification of the secondary structure elements of p7, and to the construction of a three-dimensional model of the monomer in a phospholipid bilayer. The first TM segment can be divided into an N–terminal helix (2–16) and the TM1 helix (19–33), separated by a turn involving the highly conserved G18 residue [16], [42]. TM1 is connected to the second TM segment, TM2, by a long cytosolic loop containing two fully conserved basic residues at positions 33 and 35. The TM2 helix is slightly bent due to the presence of residue P49, and the seven-residue C–terminal segment is unfolded. The overall structural motif is in keeping with that deduced from the NMR experiments reported by Cook et al. [44]–[46].

p7 oligomers have been found in both heptameric [17] and hexameric [15], [18] forms, and several hypothetical models of the p7 complex based on secondary-structure predictions have been reported [17], [18], [22], [47]. Clarke et al. [17] observed heptameric p7 complexes in low-resolution TEM images, and suggested an arrangement in which the monomers are packed in such a way that TM1 from one monomer could form favorable hydrophobic contacts with the TM2 of the next monomer. A similar heptameric model was later constructed by StGelais et al. [22]. Patargias et al. [47] modeled a hexameric complex employing structure prediction and rigid-body docking in which adjacent monomers had minimal contact. Most recently, Luik et al. [18] observed p7 hexameric complexes in short-tail DHPC (1,2-diheptanol-sn-glycero-3-phosphocholine) lipids by electron microscopy (EM), constructing a low-resolution three-dimensional density map at 16 Å resolution, which revealed a highly tilted, flower-like arrangement.

While the basic structural features of p7 are becoming gradually better understood, the conditions that lead to the assembly of a functional channel and the mechanism of ion channelling in terms of gating and selectivity remain in large measure unknown. In the work presented here, we used the monomeric p7 structure from Montserret et al. [16] to construct oligomeric models with four to seven subunits, which were evaluated via MD simulations in a hydrated POPC (1-palmitoyl-2-oleoyl-sn-glycero-3-phosphocholine) bilayer, the thickness of which resembles that of the ER membrane. In addition, we fitted our most favorable hexameric model into the flower-like EM density kindly provided by N. Zitzmann [18] and immersed the resulting structure in POPC (C16:C18) and DHPC (C7) bilayers in an effort to understand the connection between the highly-bent structure found in DHPC lipids and our more upright channel models, which would seem better suited to the ER membrane. Our work can by no means be considered an exhaustive combinatorial search of all possible p7 oligomer structures; instead, we focused on constructing and evaluating structures representative of the types of putative p7 models already suggested in the literature [17], [18], [22], [47]. In the absence of high-resolution structural data for p7, these simulations allowed us to determine likely characteristics of a functional p7 channel, including the optimal number of monomers, the possible role of certain pore-lining residues in channel gating, and the adaptability of the structure to different lipid environments. Our results reveal that p7 appears to form structurally plastic, minimalist ion channels, compatible with the coexistence of multiple oligomeric states.

## Results

### Structural properties of the oligomeric models

**Figure 1:**
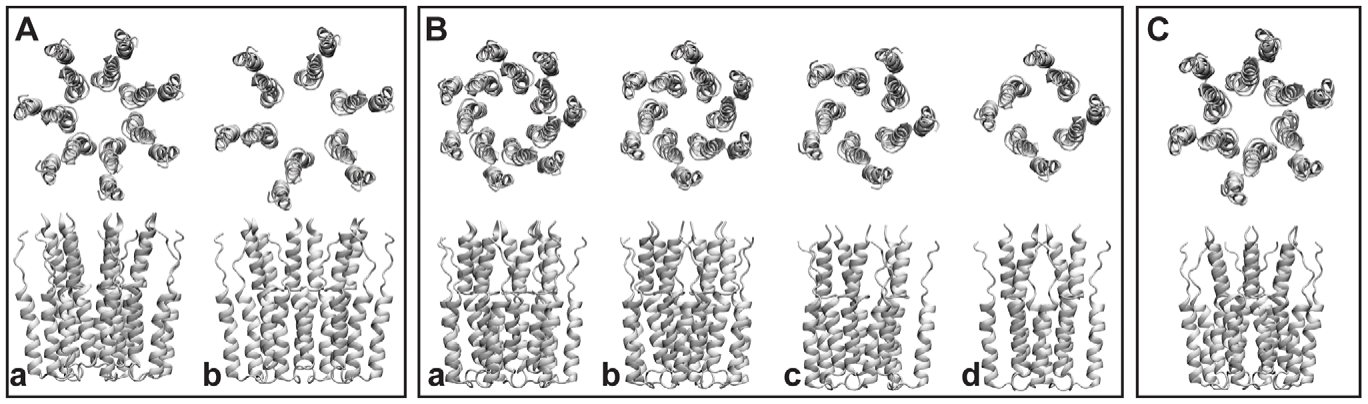
Oligomeric p7 models constructed from the NMR p7 monomer [**16**], using symmetry operations. Box A: (a) Heptamer A, (b) Hexamer A; Box B: (a) Heptamer B, (b) Hexamer B, (c) pentamer, (d) tetramer; Box C: Hexamer C. The models in box A were constructed so that the monomers extend radially away from the center, similar to the model presented earlier [47]. In contrast, the models in box B were built such that the monomers optimize the contacts between their mutual surfaces, consistent with the models described in two prior studies [17], [22]. The model in box C was built to be similar to, but have the opposite handedness of Hexamer B.

**Figure 2:**
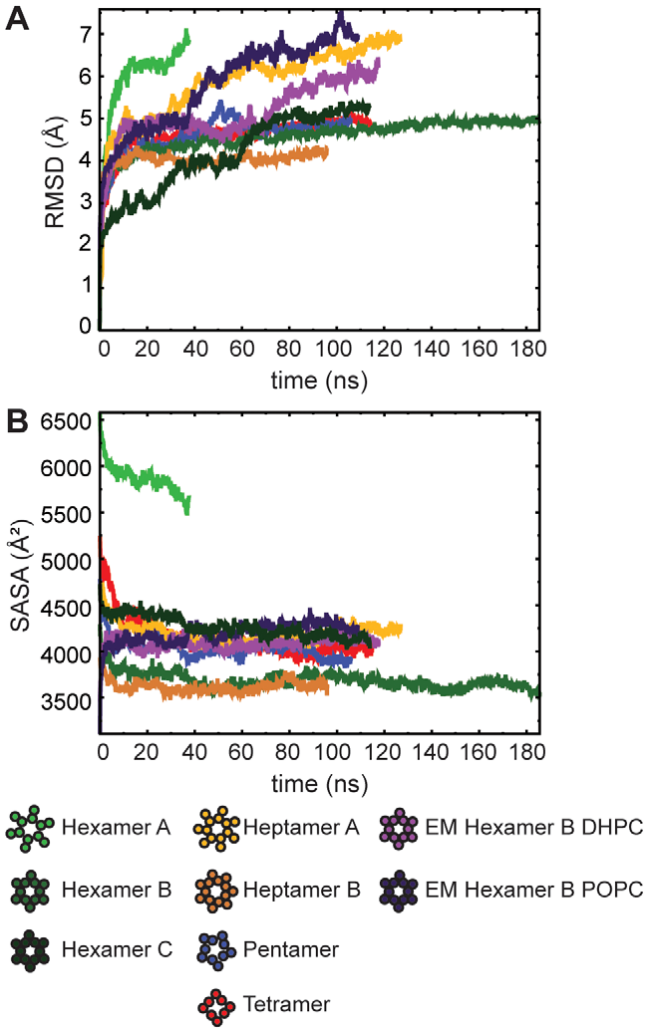
Time-evolution of the structural parameters of the oligomers. Distance root-mean square deviation (RMSD) with respect to the starting conformation of the p7 channel, computed over backbone atoms (A), and solvent-accessible surface area (SASA) per subunit (B).

Figure 1 illustrates the different p7 oligomeric models that have been constructed. The Hexamer B and Heptamer B models (Figure 1B) remained robust throughout the simulation, as demonstrated by their overall retention of structure and symmetry, as well as by the quickly plateauing distance root-mean square deviation (RMSD) with respect to the starting arrangement (see Figure 2). Noteworthily, the conformation of the different p7 subunits is similar to that of the isolated monomer when immersed in a hydrated POPC bilayer, [16], [43] but at variance with that inferred from EM densities in a thinner DHPC environment [18] (see below). Unlike Hexamer B, the Hexamer A model (Figure 1A) quickly collapsed during equilibration, as evidenced by loss of tertiary structure and partial loss of secondary structure. The Heptamer A model (Figure 1A) did not collapse, but rather began to re-arrange itself to make more inter-subunit contacts, forming a seemingly stable complex. The Hexamer C model (Figure 1C) also remained stable, but with a slightly larger RMSD compared with Hexamer B. The tetrameric and pentameric motifs also appear to be robust throughout the simulations. Although p7 has never been reported hitherto as a tetramer or a pentamer, such structures may be viable, and the possibility of their presence in the cell membrane cannot be ruled out, perhaps as intermediate stages in p7 self-assembly prior to the formation of the final hexameric or heptameric complexes.

The coexistence of multiple oligomeric forms, including those with even and odd numbers of subunits, would not be unique to p7. For example, the Vpu viroporin of HIV-1 exhibits such polymorphic behavior [48], [49], as does the antimicrobial peptide alamethicin [50]. Because the p7 monomers are not covalently bound, but instead interact primarily via van der Waals and hydrogen-bonding contacts (see Table 1 and Figure S1), there could easily be more than one oligomeric arrangement for which inter-subunit contact is sufficient to support a stable structure.

**Table 1:** Average number of inter-subunit hydrogen bonds, per subunit.

System	Avg. # hbonds
Heptamer A	0.32
Heptamer B	0.49
Hexamer A	0.27
Hexamer B	0.82
Hexamer C	0.56
Pentamer	0.31
Tetramer	0.16
EM-fitted hexamer in DHPC	0.20
EM-fitted hexamer in POPC	0.29

Inter-subunit interactions were evaluated for each of the oligomeric models. The Hexamer and Heptamer B models appear to have the best inter-subunit contact, as illustrated from the solvent-accessible surface area (SASA) per subunit, which is seen to be minimized in the Hexamer B and Heptamer B models (Figure 2B). The pentamer, tetramer, Heptamer A, and Hexamer C had similar SASAs. Analysis of inter-subunit hydrogen bonding showed that the Hexamer B model featured more hydrogen-bonding between subunits than any of the other models, even though presumably the monomers are more closely packed in the Heptamer B model (see Table 1). Hexamer C formed fewer hydrogen bonds than Hexamer B, albeit this is consistent with its larger SASA. The pentameric and tetrameric, as well as the Hexamer A and Heptamer A models involved very little hydrogen bonding. In the isolated, monomeric protein, TM1 and TM2 are, at least in part, held together by virtue of a double 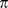
–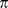 interaction resulting from the stacked F26, Y45 and W30 residues [16]. These interactions appear to be roughly preserved in the p7 oligomers (see Figure S5). Additionally, the inter-subunit interaction energies were measured for each oligomer model, and were observed to be lowest for the Hexamer B and Heptamer B models (see Figure S1). These values ought to be regarded at a qualitative level, as they ignore the entropic contribution to the self-assembly of the monomers into oligomers. Ideally, it would be desirable to determine rigorously the binding free energy associated to the formation of the latter, but this enterprise, arguably not amenable to the current capacities of MD simulations, falls beyond the scope of the present investigation.

### The central pore of the p7 oligomers

The Hexamer B, Heptamer B, pentamer and tetramer models (which are right-handed as viewed from the terminal side) were thought to be promising in terms of the residues that point towards the center of the pore, in particular, H17 and F25, which had already been proposed to face the pore, but also the hydrophilic S21 (see Montserret et al. [16] and references therein). Hexamer C, which has the opposite handedness as Hexamer B, in contrast leaves mostly hydrophobic residues facing the interior of the pore, which seems unfavorable. The initial structure of Hexamer C also leaves H17 pointing towards the body of the protein, rather than towards the central pore, though over the course of the simulation, three of the subunits rotated slightly to allow H17 minimal access to the central pore. The pore lining residues of Heptamer A are similar to those of Hexamer C. The Heptamer A model, in which the subunits initially pointed away from the center, eventually settled into a conformation with more contacts and a slight bias towards left-handedness, similar to Hexamer C. A list of pore-lining residues for each model is given in Table 2. Our simulations thus cannot rule out the possibility of left-handed p7 oligomers; however, the set of pore-lining residues seems inconsistent with existing data.

**Table 2:** List of pore-lining residues for each p7 oligomer model.

System	Pore Lining Residues
Heptamer A	TYR31, ALA28, VAL24, LEU20, HIS17 (partial), ALA16
Heptamer B	ILE32, PHE25, SER21, HIS17, SER12
Hexamer A	(collapsed)
Hexamer B	ILE32, PHE25, SER21, HIS17, VAL13/SER12
Hexamer C	TYR31, ALA27, VAL24, LEU20, HIS17 (partial), ALA16 (partial)
Pentamer	ILE32, ALA29, PHE25, SER21, GLY18 (partial), HIS17 (partial), VAL13 (partial)
Tetramer	ALA29, PHE25 (partial), PHE22 (partial), SER21, HIS17, VAL13 (partial)

**Figure 3:**
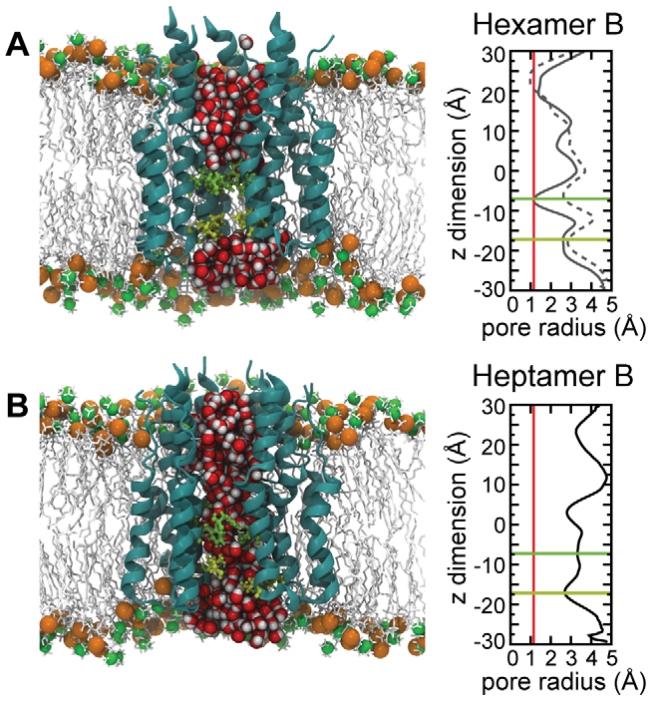
Molecular assemblies and pore-radius profiles of p7 Hexamer B (A) and Heptamer B (B) pores in a POPC bilayer. Left panels: side views of Hexamer B and Heptamer B (ribbon representation) with water molecules inside the central pore represented as van der Waals spheres (red and grey). The side chains of F25 (in green) and I32 (in yellow) are represented as sticks. The model POPC bilayer is described by means of a stick representation (light grey), with phosphorus and nitrogen atoms displayed as glossy orange and green van der Waals spheres, respectively. Right panels: radial profiles depicting the width of the pore along the longitudinal axis of the complex were calculated using the program Hole [73]. The red line is positioned at 1.4 Å, the approximate radius of a water molecule. The green and yellow lines mark the approximate positions of F25 and I32, respectively. In the Hexamer B plot (A), the solid line represents the radial profile before the opening of the pore and highlights a constriction at the level of F25. The dotted line denotes the radial profile after the pore has opened (the protrusion near z = 25 Å is the result of association between the N-terminal tails of the six subunits in the solvent below the membrane, and does not represent a blockage of the pore).

With the exceptions of Hexamer B and Hexamer C, all channel models formed a continuously open solvent-accessible pore, as illustrated by Heptamer B in Figure 3B. In the case of Hexamer B (see Figure 3A), the pore was initially sealed, but later opened, allowing water molecules to flow through for the remainder of the simulation. When sealed, the pore of Hexamer B was blocked in two places by rings of adjacent hydrophobic residue side chains, thereby forming an energetic barrier to water permeation. This constriction is manifested also in a discontinuity in the electrostatic potential within the channel (see Figure S2). One seal was found at the level of residue F25, already hypothesized to play such a role [16], with a second seal at the level of I32, which was not yet identified to be a putative pore lining residue. The F25 barrier is revealed in the pore-radius profile of Hexamer B (see Figure 3A), which depicts the width of the pore along the longitudinal axis of the complex. In the first 65 ns of the simulation, the protrusion of the F25 ring constricts the pore to a radius smaller than that of a water molecule (solid line and red line, respectively, Figure 3A). The barrier formed by the F25 side chains recedes when the pore opens, allowing water permeation (dotted line, Figure 3A). In contrast, the pore-radius profiles characterizing the other models reveal no constrictions at the level of F25 narrow enough to preclude water diffusion (Figure 3B and Figure S3). The I32 side chains are not bulky enough to block the channel; possibly they prevent the passage of water by withholding hydrogen-bonding partners. A similar behavior was noted in MD simulations of M2 due to a ring of valine residues [51]. In the present case, the opening of the channel was precipitated by the interaction of water molecules with the F25 side chains. Random fluctuations of the F25 side chains eventually allowed a water molecule to slip through, after which the pore opened quickly (see Video S2).

The Hexamer C model was also initially sealed, this time at the level of Y31 and L20. We did not observe spontaneous opening of the central pore during the simulation. In order to also sample the open conformation of the pore, we briefly applied an electric field in the simulation, which resulted in rearrangement of the side chains of the pore-lining residues sufficient to open the pore. Hexamer C was then allowed to equilibrate in the open conformation.

### Conductance measurements

Conductance calculations were performed for the Heptamer A, Heptamer B, and Hexamer B models by applying a constant electric field to the MD simulation and analyzing the subsequent flux of ions through the channel, as described in Materials and Methods, using 250 mM KCl. The results of these calculations are summarized in Table 3. Montserret et al. [16] reported conductance measurements of 22 pS at 60 mV and 41 pS at 140 mV. Ion translocation failed to occur in simulations with potential differences of 60 mV and 140 mV; it was therefore necessary to apply higher voltages and interpolate the resulting measurements under the assumption that conductance scales linearly with voltage.

**Table 3:** Conductance simulations performed.

System	Voltage	Time	Conductance	Uncertainty	Equiv. at 60 mV	Equiv. at 140 mV
Heptamer A	1500 mV	62 ns	183.5 pS	29.3 pS	7.3 pS	17.1 pS
Heptamer B	1500 mV	81 ns	130.4 pS	19.8 pS	5.2 pS	12.2 pS
Hexamer B	1500 mV	56 ns	236.2 pS	33.8 pS	9.4 pS	22.0 pS

Simulation of Hexamer B at 1500 mV yielded a conductance measurement of 236.2 pS, which, interpolated to 60 mV and 140 mV, would give 9.4 pS and 22 pS, respectively. The Heptamer B model gave a conductance value of 130.4 pS at 1500 mV, which would give 5.2 pS and 12.2 pS at 60 mV and 140 mV, respectively, and Heptamer A yielded 183.5 pS at 1500 mV, giving 7.3 pS and 17.1 pS at 60 mV and 140 mV. That the simulations give conductance values with the correct order of magnitude is encouraging. We do not, however, expect the simulated conductance values to match the specific values obtained experimentally. In experimental conductance measurements, it is impossible to know the proportion of heptamers to hexamers in the sample, and the resulting measurement represents an ensemble average of the action of all the oligomers in the sample. By contrast, MD simulations can only report the behavior of one specific structure. Additionally, the treatment of ions in MD with the standard, non-polarizable CHARMM force field is imperfect, and yields behaviors and diffusion constants which differ slightly from experimental values [52], [53].

One intriguing feature of the present set of conductance calculations is the difficulty of reproducing the expected ion selectivity, which is somewhat higher for cations according to experiment [16], [43]. Discrepant ion selectivities between simulations and expriment are not novel [54], [55]. In the present case, it is believed to stem from clogging of the pore entrance by chloride ions interacting with the titratable K33 and R35 residues of the loop regions. Over the time scale of the simulations, unbinding events of the anions are too scarce to permit diffusion of potassium ions through the pore. Yet, if the positively charged residues at positions 33 and 35 are replaced by glutamine, the experimentally observed ion selectivity is recovered (see Video S4), hence suggesting that either the present trajectories are too short, or that ion parametrization is suboptimal [53].

### Reconciling the models with EM densities

**Figure 4:**
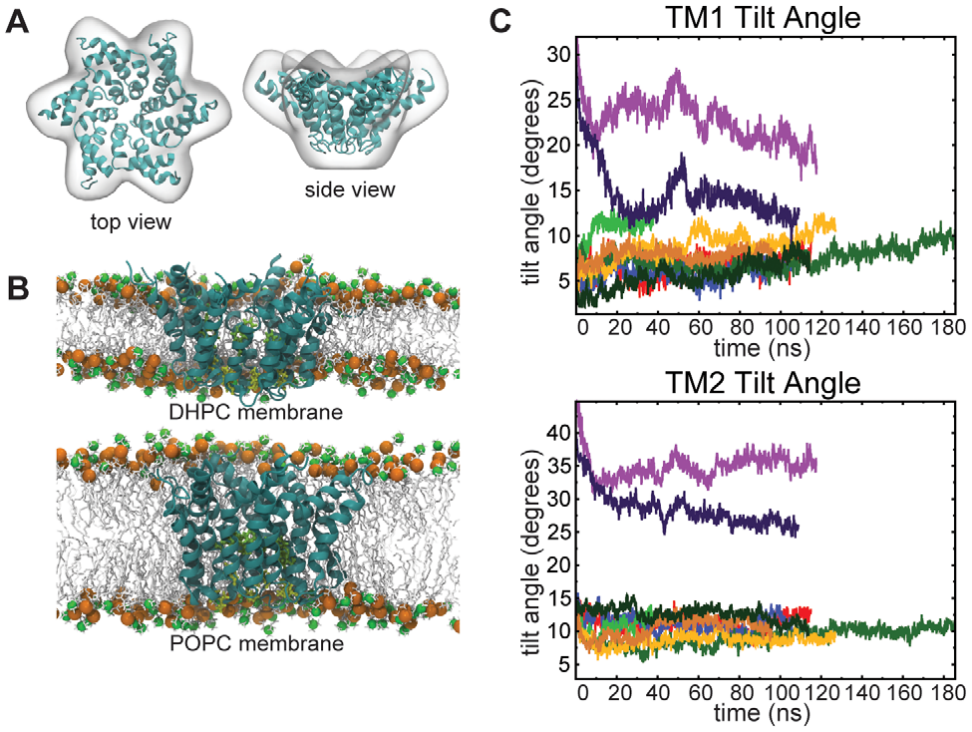
Conformation of the p7 hexamer in short– and long–tail lipid environments. (A) Hexamer B model (ribbon representation) fitted into the EM envelope of Luik et al [18]. (B) Side views of the EM-fitted hexamer model in the DHPC and the POPC lipid bilayers (for details and color code, see legend of Figure 3). (C) Tilt angles of TM1 and TM2 helices calculated for all models (for color code of oligomers, see legend of Figure 2). Relaxation of the model placed in POPC towards an upright conformation akin to that of Hexamer B is recognized through lower values of the TM1 and TM2 tilt angles.

In contrast with the flower-like EM model of the p7 hexamer reported by Luik et al. [18], the Hexamer B model that we propose to represent a functional p7 channel appears to be a cylindrical, upright complex in the membrane. This discrepancy could be due to the size difference of the phospholipid hydrophobic chain, which is very short in the DHPC environment used in EM studies (C7), compared to that of POPC used in MD simulations (C16:C18). To test this hypothesis, we drove the p7 Hexamer B model into the EM envelope of the p7 hexamer using the molecular dynamics flexible fitting (MDFF) algorithm (see Video S1). In recent years, MDFF has been used successfully for structure determination with higher-resolution maps [56]–[59]. Yet, structures obtained using lower-resolution data, such as the 16-Å p7 map, ought to be viewed as suggestive rather than as representing an accurate native structure. In fact, there is some variation in the final structure based on the original orientation of the structure and the force constants utilized to fit the Hexamer B into the EM map (see Figure S4). Once driven into the EM envelope (Figure 4A) and equilibrated via MD in the thin DHPC environment (Figure 4B), the simulated structure largely retains its original bent conformation, in agreement with the experimental observations of Luik et al. [18]. Conversely, if placed in a POPC environment, which would more closely resemble the ER membrane, the helices begin to straighten up as the structure progressively evolves towards a more upright conformation, akin to that of Hexamer B described above (see Video S3). The tilt of the inner and outer helices of the structure is displayed in Figure 4C. Put together, these results illuminate the structural plasticity of the p7 monomer in an oligomeric context, and, hence, its adaptability to the membrane bilayer thickness.

## Discussion

In this article, we have described the construction of several model p7 oligomers with four to seven subunits. We find that all oligomerization states investigated form structurally stable ion channels, provided that neighboring subunits come into close contact. Oligomers with six or seven subunits display a slight advantage over oligomers with four or five subunits in terms of inter-subunit interactions, consistent with the prevalence of hexameric and heptameric complexes observed in analytical centrifugation experiments [16] and reported by others [15], [17], [18]. Oligomeric models constructed for alternate genotypes of p7 were also found to be stable in preliminary simulations (data not shown), thereby further reinforcing the view of robust channels. In our simulations, we observed that the heptameric pore was constantly accessible to the solvent, whereas the hexameric pore was blocked for part of the trajectory. This behavior is suggestive of a picture of the cell membrane in which transiently open p7 hexamers might be accompanied by always-open p7 heptamers. Each of the models were constructed from the p7 monomer described earlier [16], resulting in cylindrical oligomers, in contrast to the corolla-shaped complex observed by Luik et al. [18]. The cylindrical hexamer can, however, be fitted into the flower-like EM envelope. p7 thus appears to be sufficiently flexible to adapt to varying lipid environments, like DHPC and POPC, naturally resulting in a highly tilted structure in short-tail lipids, and a more upright conformation in long-tail lipids, the latter being compatible with the thickness of the ER membrane. We note that this structural flexibility is shared by other viroporins such as Vpu of HIV, which has been reported to adapt to the lipid environment via kinking rather than forcing the lipid environment to adjust [60]. Very recently, it has been demonstrated that changes in lipid composition modify the ion-channel activity of p7, suggesting that p7 can indeed rearrange in response to changes in the lipid environment [61]. Altogether, our findings allow us to conclude that p7 forms flexible and minimalist, yet robust, functional ion channels.

Efforts to resolve the complete structure of native viroporins with an accuracy adequate to determine their ion-channel mechanisms face particular challenges. Aside from the intrinsic difficulties in preparing large amounts of purified membrane protein, the structural flexibility and polymorphism of viroporins deeply complicate experimental investigations, as exemplified in the case of Vpu for which the numerous studies published over the last 15 years have not yielded a consensus on the mechanisms underlying its function. These challenges explain in large measure why efforts to disentangle the mechanisms that underlie the formation and the function of viral ion channels have remained hitherto rather scarce. Computational studies of viroporins are burdened with skepticism that useful information can be inferred from simulations relying on a modeled structure. However, in the context of the HCV p7 viroporin as an attractive therapeutic target, MD simulations based on three-dimensional constructs offer a way to yield novel insights in a field where high-resolution data is lacking. The authors admit that even over ample time scales, successful MD simulations are not enough to prove the accuracy of a model; however, they remain a useful tool to probe the viability of a model, detect flaws in its construction and confront hypotheses put forth by experiments. In the instance of p7 oligomeric channels, the wealth of structural information accrued in recent years, particularly on the monomeric state of the protein, constitutes a solid base for the design and refinement of the p7 complexes described herein.

The observation that at least one oligomeric construct of each class examined here was structurally stable in simulation suggests that complexes with four to seven subunits could coexist in the cell membrane. Reported data [15]–[18], however, reveal that p7 is largely observed as hexa- and heptameric complexes. It is possible that tetra- and pentameric complexes can form, but are in some way unfavorable or less functional as ion channels compared to hexa- or heptameric complexes. In addition, hexa- and heptameric complexes may be more favorable energetically, such that tetra- or pentameric complexes would organize only transiently, as intermediates in the formation of hexa- or heptameric complexes. This is in agreement with the notion that, due to their small size, viroporins most likely self-assemble to form a pore in the membrane [37]. In the case of p7, this notion is supported by the spontaneous assembly of p7 oligomers exhibiting active and appreciably selective cation channeling when reconstituted with lipids [15] [16 [19]–[22].

Although concerted organization in an all-or-nothing step cannot be ruled out, our results suggest that p7 monomers assemble sequentially. An important consequence of such a mechanism relates to the regulation of p7 release in the HCV life cycle. It is unlikely that p7 monomers are released during the processing of the HCV polyprotein at the ER membrane since the spontaneous formation of active p7 ion channels could stress the ER membrane, ultimately leading to cell apoptosis [62]. Given that no feedback mechanism regulating p7 activity has yet been identified, it is reasonable to suggest that the release of free p7 must be regulated upstream. p7 has been reported to interact with various HCV proteins including core, E1, E2, and NS2 [24], [28]–[31], [33]. It is possible that p7 is retained in an inactive state via its interactions with other viral factors, and is later released when it is needed for HCV particle assembly and/or egress. A similar hypothesis has been put forth in the case of the HIV viroporin, Vpu, which can engage in protein-protein interactions, but can also self-assemble to form channels or pores [37]. Because of its central organizing role in HCV assembly, it is tempting to speculate that NS2 is involved in the orchestration of p7 release and assembly [Bibr pcbi.1002702-Jirasko1] [28]–[Bibr pcbi.1002702-Popescu1] [31]. Following this hypothesis, one can assume the existence of a specific molecular mechanism allowing the putative p7-NS2 complex to dissociate at the critical stage of viral-particle formation and egress. Further, one cannot exclude that variation in the thickness between the ER and the export-vesicle membranes could play a role in the regulation of p7 oligomerization, as well as in ion-channel activity, just as the composition of the lipid membrane appears to do [61].

Compared with selective ion channels of eukaryotic cells like, for instance, voltage-gated potassium channels, which exhibit specific pore-lining motifs [63], the p7 channel appears to have a minimalist channel architecture, as generally observed in viroporins [37]. It is solely held together through non-covalent, inter-monomer interactions, and tends to form a stable pore open to the solvent. The transient nature of the pore in the Hexamer B model suggests the possibility of gating via minimal conformational change. Noteworthily, none of the p7 hydrophilic pore-lining residues studied by mutagenesis, which display substantial intra- and inter-genotype variability, appears to be really essential for p7 ion channeling in vitro (discussed in [16]). Our study indicates a role for hydrophobic residues at or near positions 25 and 32 in gating the p7 channel. The involvement of F25 was already suggested by the hyperactivity of channels in which F25 and neighboring F22 and F26 were replaced by alanines [22], as well as its role in conferring resistance to alkylated imino-sugars [10], but the significance of I32 has yet to be explored. While there is some variation in sequence at these positions across the multiple genotypes of p7, there seem always to be bulky hydrophobic residues at positions 32 and 24 or 25. The presence of these hydrophobic barriers prompts a scenario in which gating could be promoted by small movements of the 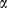-helical segments (see the earlier discussion in Montserret et al. [16]). Together with the fact that p7 is only weakly ion selective, it is possible that hydrophilic pore-lining residues would be all that is required to attract and conduct ions across the channel. All these features, shared among most viroporins, are consistent with the notion that the mechanism of gating of viroporins in general [37], and of p7 in particular, is rather minimalist. These findings have important implications for the development of drugs aimed at blocking p7 activity. Targeting of pore lining-residues could, indeed, be vain and/or rapidly induce viral resistance, as suggested by the large natural amino acid variability. In contrast, compounds targeting strictly conserved residues essential for p7 assembly could inhibit oligomer formation and are expected to be more promising.

## Materials and Methods

### Construction of the molecular assemblies

The p7 oligomeric models (genotype 1b, strain HCV-J, accession number D90208) were constructed by applying the appropriate symmetry operations to the model of the p7 monomer determined by NMR and MD, as described by Montserret et al. [16]. The homogeneity of the NMR signals, measured for p7 reconstituted with phospholipids [44], mirrors that of the structure of the individual p7 subunits, and further supports the notion of symmetrically-arranged p7 oligomers.

Insofar as the hexamer and the heptamer are concerned, two types of oligomeric states were constructed — one in which the monomers extend radially away from the center and have minimal contact with one another (referred to as Hexamer A and Heptamer A, Figure 1A), consistent with the model put forth by Patargias et al. [47], and one in which the monomers are rotated such that adjacent subunits make better contact (referred to as Hexamer B and Heptamer B, Figure 1B), in line with the models described in [17], [22]. The tetrameric and pentameric constructs followed the pattern of Hexamer B and Heptamer B. Finally, an additional hexameric model (referred to as Hexamer C) was constructed, which was similar in arrangement to Hexamer B, but with opposite handedness. All models are depicted in Figure 1.

The Hexamer B p7 model was fitted into the EM map from [18] employing the molecular-dynamics flexible-fitting (MDFF) algorithm [64]. The MDFF method utilizes the EM map to create an external potential, which drives the atoms towards regions of higher density. During the fitting process, geometrical restraints are enforced on the dihedral angles to preserve the secondary structure of the protein. In fitting p7 into the EM map, additional forces were applied to maintain the six-fold symmetry of the model.

### Molecular dynamics simulations

In each of the p7-membrane systems, an all-atom representation was used for protein, water and ions, whereas the united atom lipid model described in [65] was used for the lipids. All simulations were performed using the MD program NAMD [66] and the CHARMM27 force field with CMAP corrections [67], [68]. The equations of motion were integrated with a multiple time-stepping algorithm [69], [70] in which bonded interactions were evaluated every 2 fs, short-range non-bonded interactions every 2 fs, and long-range electrostatics interactions every 4 fs. Short-range non-bonded interactions were truncated smoothly with a spherical cutoff radius of 12 Å, and a switching distance of 10 Å. Periodic boundary conditions were assumed. Long-range electrostatic interactions were computed employing the particle mesh Ewald (PME) method [71], with a grid point density of approximately 1/Å^3^. Temperature and pressure were maintained at 300 K and 1 bar, respectively, using Langevin dynamics with a friction coefficient of 1 ps^−1^ and the Langevin piston method [72].

Table 4 lists all simulations performed. In the tetramer, pentamer, hexamer and heptamer simulations, the p7 model was placed in a fully hydrated, thermalized POPC membrane, and restrained for the first few nanoseconds to enhance lipid packing around the protein. The restraints were then released and the protein was allowed to equilibrate in the isobaric-isothermal ensemble for the times listed in Table 4. The upright Hexamer B model was fitted into the EM map, and the resulting structure was then placed in POPC and DHPC membranes. Again, motion of the protein was initially restricted to improve lipid packing; after this stage, additional restraints were applied to preserve the secondary structure and symmetry of the complex. These restraints facilitated a gentle release from the EM-fitted structure. Ultimately, all restraints were removed and the protein was allowed to equilibrate in the isobaric-isothermal ensemble.

**Table 4:** List of the constructed p7 oligomers and MD simulations performed.

System	Time (ns)	Atoms	Lipid	Dimensions (Å^3^)
Heptamer A	126	51,797	POPC	111×113×90
Heptamer B	96	49,303	POPC	111×113×90
Hexamer A	38	48,110	POPC	111×113×90
Hexamer B	185	48,626	POPC	111×113×90
Hexamer C	115	48,248	POPC	94×81×74
Pentamer	106	48,275	POPC	111×113×90
Tetramer	115	48,075	POPC	111×113×90
EM-fitted Hexamer B	109	83,052	POPC	117×127×90
EM-fitted Hexamer B	118	102,092	DHPC	128×137×75

### Conductance calculations

Conductance measurements can be obtained from MD simulations by applying a constant electric field to the system in the 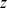 direction. The ionic current is given by
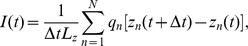
where 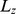 is the 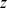 dimension of the periodic box, 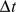 is the chosen time interval (1 ps in our calculations), 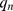 and 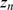 are the charge and 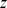 coordinate of atom 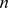. The sum runs over all of the atoms enclosed in a defined region of interest (usually the interior of the pore) at time 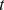. An average current is calculated from the instantaneous currents measured over the course of the conductance simulation. The conductance is then given by 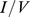. The uncertainty in the conductance is approximated, assuming Poisson statistics of independent events, as 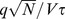, where 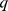 is the ion charge, 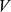 is the applied voltage, and 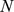 is the total number of ion translocation events observed in simulation time 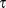. An ion concentration of 250 mM KCl was used.

## Supporting Information

Figure S1 Interaction energies per subunit, averaged over the trajectory. Heptamer B and Hexamer B display lower total interaction energies. The differences in interaction energies between the models is clearly dominated by the van der Waals component.
(TIF)

Figure S2 Electrostatic potential maps of (A) Heptamer B (B) Hexamer B before opening of pore (C) Hexamer B after opening of pore. The potential map of Hexamer B before the opening of the central pore shows a barrier between the interior of the pore and the solvent; the maps of Heptamer B and of Hexamer B after the opening of the pore show that the interior of the pore is accessible to solvent.
(TIF)

Figure S3 Radial profiles of each of the p7 models, calculated using HOLE. The green and yellow lines mark the positions of F25 and I32, respectively. In the Hexamer C plot, L20 and Y31 are also marked in purple and blue, respectively. In the Hexamer B and Hexamer C plots, the solid black profile represents the model before the opening of the pore, and the dashed gray profile represents the model afterwards.
(TIF)

Figure S4A variety of models obtained by driving the upright Hexamer B model into the 16 Å EM map, as determined by differences in starting conditions (in this case the rotation angle θ about the central axis) and force constants (f and g) used in the MDFF algorithm. It was clear that the model fit best into the map at 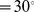 but the authors were curious how the method would handle initial configurations that were rotated away from the correct orientation. The force constant f determines the strength of the symmetry constraints, while g determines the strength of the force driving the model into the EM map. The structures obtained from higher force constants tended to be more tilted than those obtained with lower force constants, even when MDFF was applied to the latter for considerably longer times. However, the larger force constants also tended to result in more helical distortion. We chose a model (highlighted above) which we felt represented a compromise between the conflicting goals of achieving a highly-bent corolla-like shape and not introducing excessive distortion.(TIF)

Figure S5Angular distribution for the 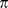-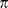 stacking interaction of residues F26 and Y45, and W30 and Y45, for each of the oligomeric p7 models. 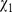 and 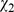 are the angles formed by the normal n_1_ to the plane of the first aromatic ring and the vector u_12_ connecting the centroid of the two interacting aromatic rings, and by the normal n_2_ to the plane of the second aromatic ring and vector u_12_. Combinations of 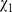 and 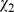 of approximately 0 or 180° correspond to 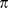-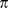 stacked motifs of the aromatic side chains. The clustering of data points around the four corners of the graph show that the stacking interactions are roughly preserved in the oligomeric models.(TIF)

Video S1 Video showing the upright Hexamer B model being driven into the EM density using the MDFF method.
(MPG)

Video S2 Video showing the simulation trajectory for the Hexamer B model; the pore opens during the simulation when the mutual interactions of the F25 side chains and I32 side chains are broken.
(MPG)

Video S3 Video showing the trajectory for the EM-fitted Hexamer B model in a POPC bilayer; over the course of the trajectory, the helices begin to straighten as a result of hydrophobic mismatch.
(MPG)

Video S4 Video showing the trajectory for an additional conductance simulation done with a K33Q-R35Q mutated Hexamer B model. The applied voltage was 1500 mV, and the system was simulated for 84 ns. Without the positively charged K33 and R35 blocking the entrance to the channel, potassium ions were able to enter and pass through the channel, and a cation selectivity, as seen in experiments, was observed. However, only a few ions passed through the channel over the course of the simulation, leading to a very small measured conductance value. This suggests that either our simulations are too short to reproduce the order of magnitude of the conductances measured in experiments, or that the ion parametrization is suboptimal.
(MPG)
