# Supplementary figures and images for: The p7 Protein of Hepatitis C Virus Forms Structurally Plastic, Minimalist Ion Channels
Source: PLoS Comput Biol. 2012 Sep 20;8(9):e1002702. doi: 10.1371/journal.pcbi.1002702 (PMC3447957; doi:10.1371/journal.pcbi.1002702)

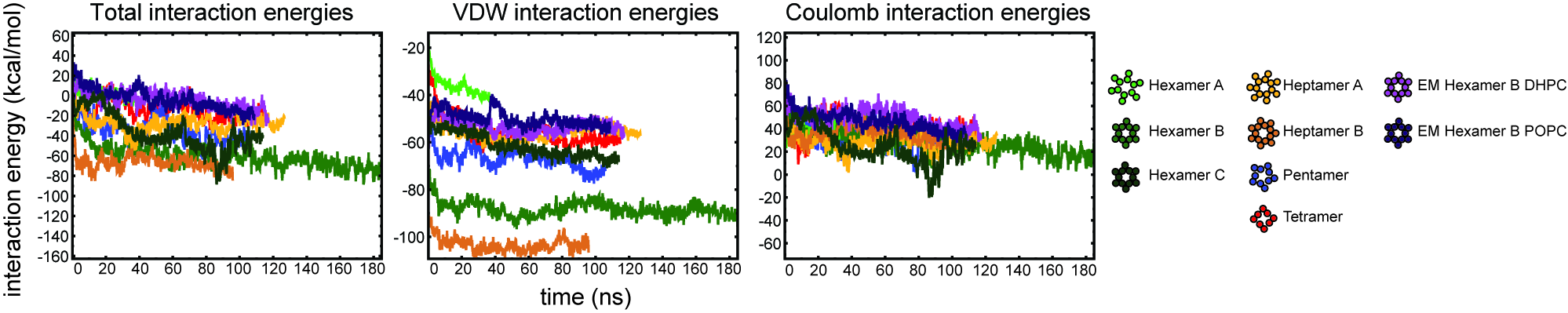

Supplement: Figure S1 — Interaction energies per subunit, averaged over the trajectory. Heptamer B and Hexamer B display lower total interaction energies. The differences in interaction energies between the models is clearly dominated by the van der Waals component. (TIF) [file pcbi.1002702.s001.tif]

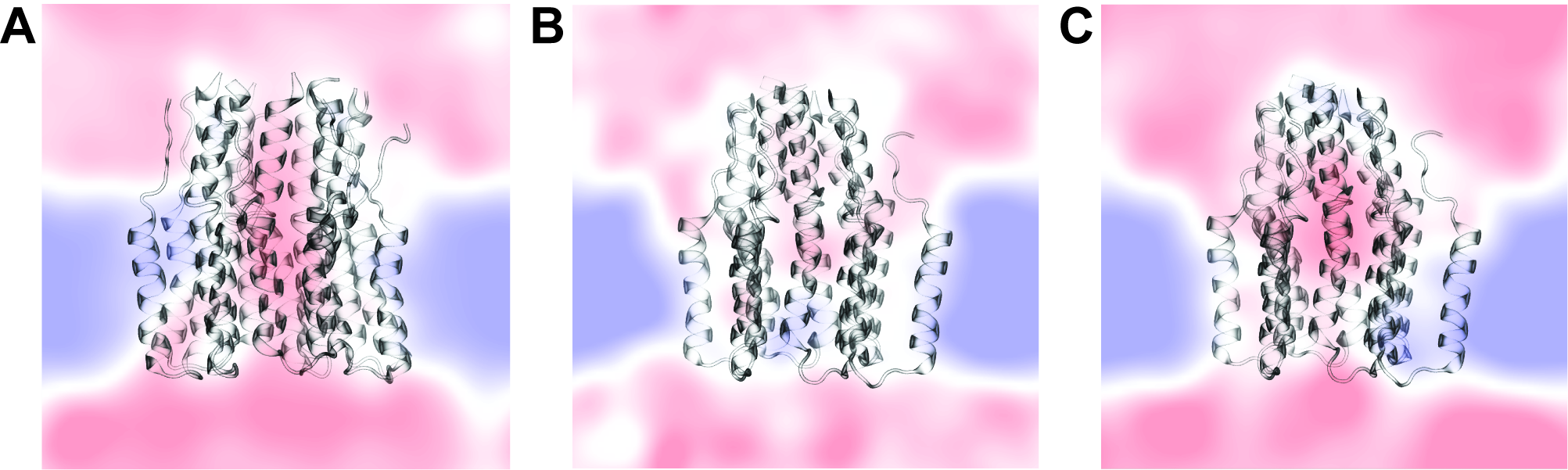

Supplement: Figure S2 — Electrostatic potential maps of (A) Heptamer B (B) Hexamer B before opening of pore (C) Hexamer B after opening of pore. The potential map of Hexamer B before the opening of the central pore shows a barrier between the interior of the pore and the solvent; the maps of Heptamer B and of Hexamer B after the opening of the pore show that the interior of the pore is accessible to solvent. (TIF) [file pcbi.1002702.s002.tif]

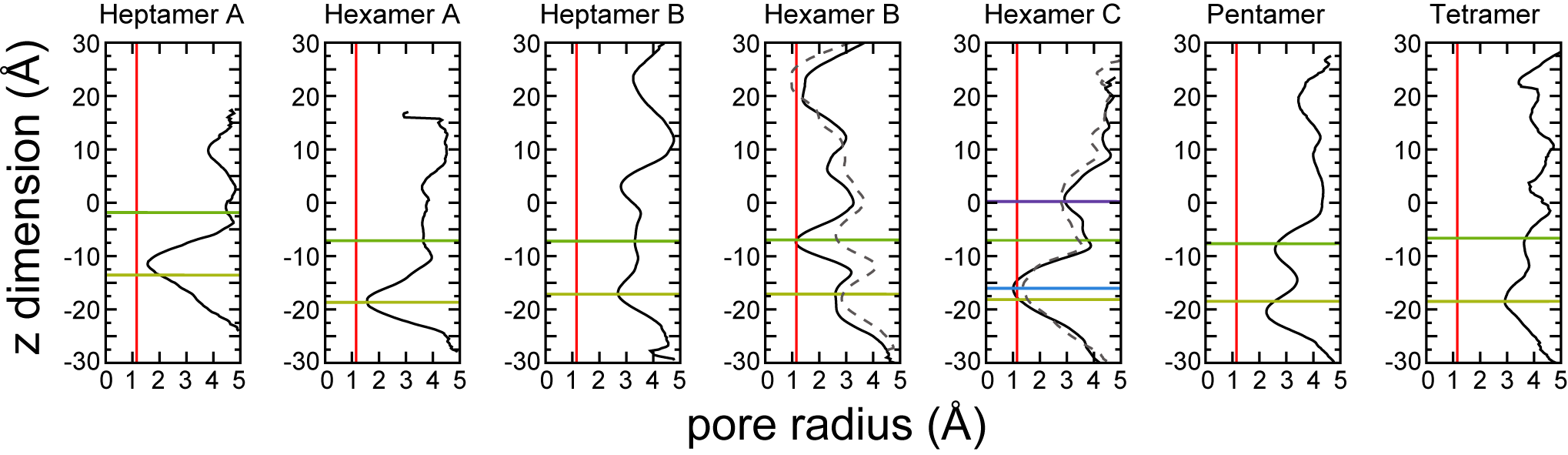

Supplement: Figure S3 — Radial profiles of each of the p7 models, calculated using HOLE. The green and yellow lines mark the positions of F25 and I32, respectively. In the Hexamer C plot, L20 and Y31 are also marked in purple and blue, respectively. In the Hexamer B and Hexamer C plots, the solid black profile represents the model before the opening of the pore, and the dashed gray profile represents the model afterwards. (TIF) [file pcbi.1002702.s003.tif]

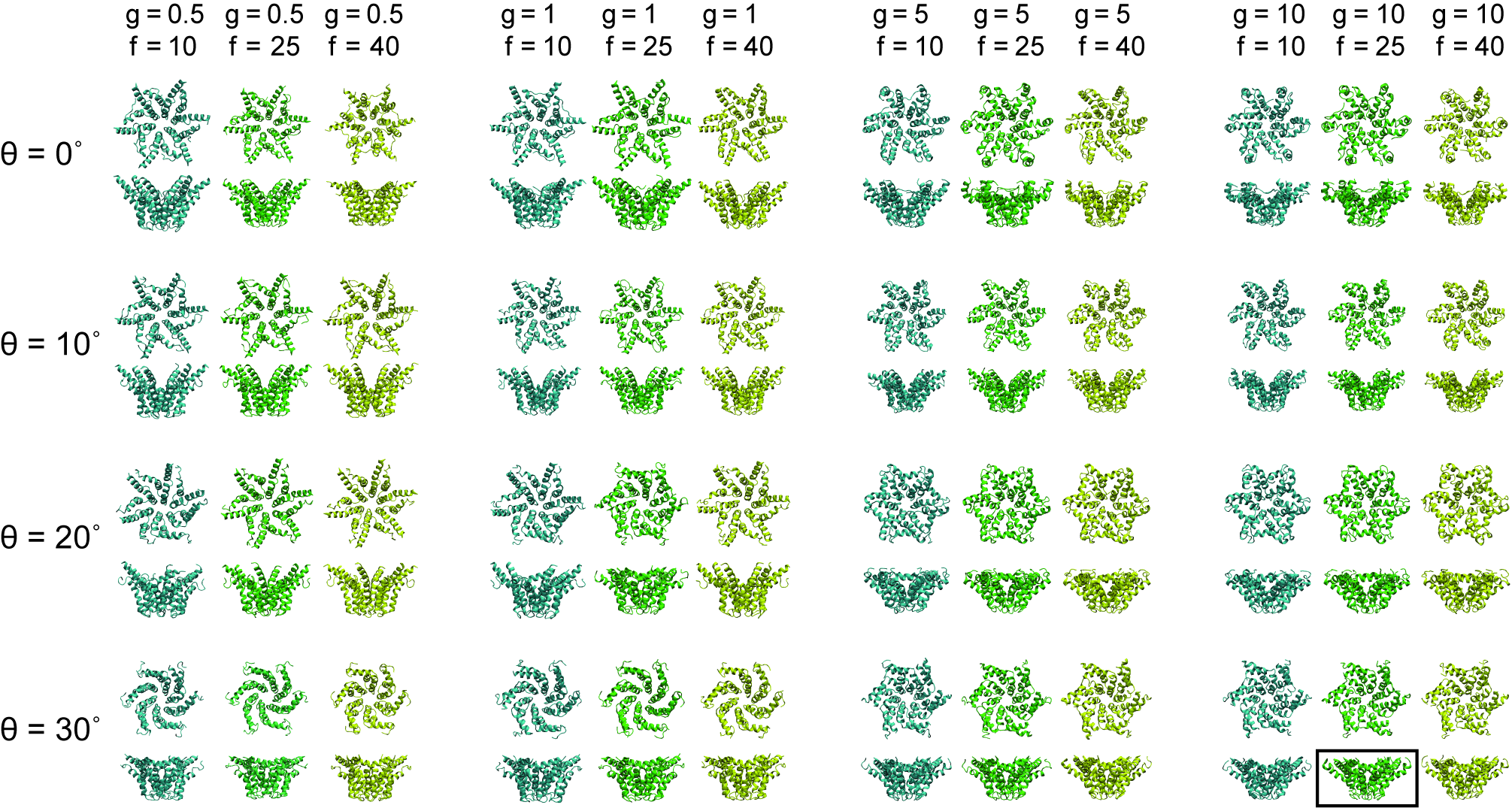

Supplement: Figure S4 — A variety of models obtained by driving the upright Hexamer B model into the 16 Å EM map, as determined by differences in starting conditions (in this case the rotation angle θ about the central axis) and force constants (f and g) used in the MDFF algorithm. It was clear that the model fit best into the map at but the authors were curious how the method would handle initial configurations that were rotated away from the correct orientation. The force constant f determines the strength of the symmetry constraints, while g determines the strength of the force driving the model into the EM map. The structures obtained from higher force constants tended to be more tilted than those obtained with lower force constants, even when MDFF was applied to the latter for considerably longer times. However, the larger force constants also tended to result in more helical distortion. We chose a model (highlighted above) which we felt represented a compromise between the conflicting goals of achieving a highly-bent corolla-like shape and not introducing excessive distortion. (TIF) [file pcbi.1002702.s004.tif]

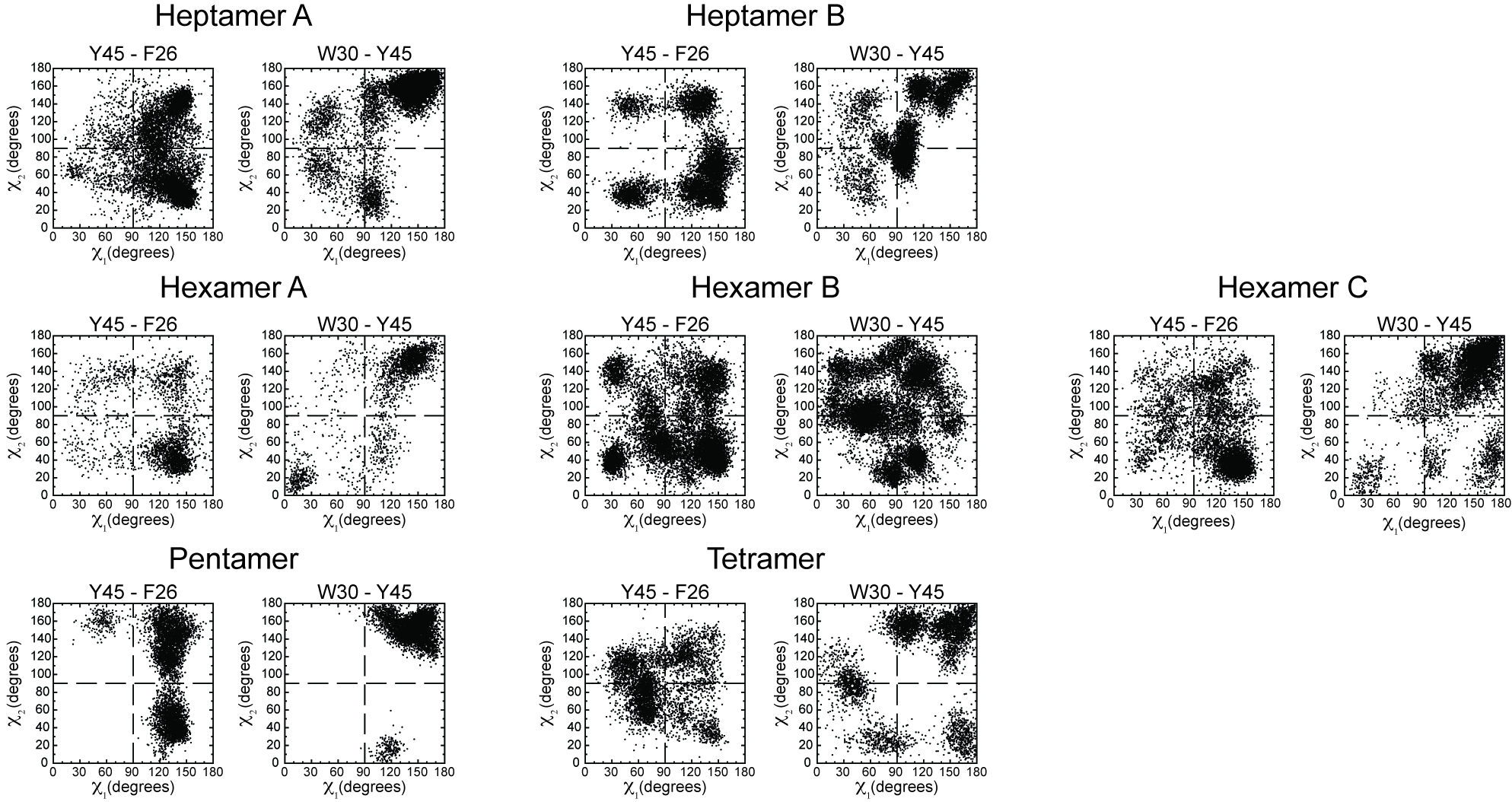

Supplement: Figure S5 — Angular distribution for the - stacking interaction of residues F26 and Y45, and W30 and Y45, for each of the oligomeric p7 models. and are the angles formed by the normal n1 to the plane of the first aromatic ring and the vector u12 connecting the centroid of the two interacting aromatic rings, and by the normal n2 to the plane of the second aromatic ring and vector u12. Combinations of and of approximately 0 or 180° correspond to - stacked motifs of the aromatic side chains. The clustering of data points around the four corners of the graph show that the stacking interactions are roughly preserved in the oligomeric models. (TIF) [file pcbi.1002702.s005.tif]
